# Remembering faces with emotional expressions

**DOI:** 10.3389/fpsyg.2014.01439

**Published:** 2014-12-10

**Authors:** Chang Hong Liu, Wenfeng Chen, James Ward

**Affiliations:** ^1^Department of Psychology, Bournemouth University, Poole, UK; ^2^State Key Laboratory of Brain and Cognitive Science, Institute of Psychology, Chinese Academy of Sciences, Beijing, China; ^3^Department of Computer Science, University of Hull, Hull, UK

**Keywords:** facial expression, transfer of expression training, identity recognition, emotion, memory

## Abstract

It is known that happy faces create more robust identity recognition memory than faces with some other expressions. However, this advantage was not verified against all basic expressions. Moreover, no research has assessed whether similar differences also exist among other expressions. To tackle these questions, we compared the effects of six basic emotional expressions on recognition memory using a standard old/new recognition task. The experiment also examined whether exposure to different emotional expressions at training creates variable effects on transfer of the trained faces to a new/neutral expression. Our results suggest that happy faces produced better identity recognition relative to disgusted faces, regardless of whether they were tested in the same image or a new image displaying a neutral expression. None of the other emotional expressions created measurable advantage for recognition memory. Overall, our data lend further support for the happy face advantage for long-term recognition memory. However, our detailed analyses also show that the advantage of happy expression on identity recognition may not be equally discernible from all other emotional expressions.

## INTRODUCTION

Happy faces seem to enjoy a special place in long-term memory. Participants are more likely to recognize a person if the person’s face was shown with a happy expression in a previous encounter. The identity of a happy face is remembered and identified more accurately compared to faces with a number of expressions, including surprise, anger, fear, and neutral expressions (Endo et al., 1992; Baudouin et al., 2000; Kaufmann and Schweinberger, 2004; Gallegos and Tranel, 2005; Shimamura et al., 2006; D’Argembeau and Van der Linden, 2007, 2011). However, not all basic emotional expressions have been compared to the happy expression in the identity recognition literature. For example, disgust is missing from the list. This makes it hard to conclude that the happy expression has a special advantage for long-term face identity memory over all other basic expressions. Moreover, because the basic expressions were rarely compared in a single study, it is difficult to ascertain whether some expressions other than happiness also have a similar facilitating effect on face memory.

Despite numerous reports, the happy-face advantage is far from clear-cut. In fact, there are several notable contradictory findings (Johansson et al., 2004; Sergerie et al., 2005, 2010; Righi et al., 2012), where memory for faces with negative expressions was better than with a positive or neutral expression.

One potential cause of the discrepancy may be the way the face stimuli were manipulated (Chen et al., 2011). Since physically distinctive faces are easier to discriminate than more typical faces (Valentine, 1991), it is critical to compare the effects of the expressions on the same face identities. The present study was an attempt to control identity differences while comparing the effects of all six basic emotional expressions on face recognition memory.

The relationship between identity and expression processing has been hotly debated since Bruce and Young’s (1986) seminal work (see a review by Calder and Young, 2005). The central issue of the debate is whether facial identity and expression are processed via two independent routes. Effects of happy expression on identity recognition have contributed to this debate by providing supporting evidence for the hypothesis that identity processing is not independent of expression processing. Recent literature appears to show that facial expressions automatically modulate the encoding of facial identity in memory (D’Argembeau et al., 2003; D’Argembeau and Van der Linden, 2007). The happy face advantage may be a result of this. It means that appraisal of facial expression accompanies identity encoding. The nature of the appraisal is not well understood. However, conjectures that lead to different predictions can be made. One possibility is that the emotional signal is classified according to the valence of an expression. According to this hypothesis, different categories of expression are reduced to a single dimension that measures valence along the axis of positive and negative polarities. Encoding of facial identity may be simply modulated by emotional valence. This predicts a positive correlation between valence of face stimuli at training and subsequent recognition performance. That is, the more positive the valence of an expression at training, the better the recognition memory of the studied face. This predicts best performance for faces trained in a happy expression, followed by those trained in a neutral expression, which may be in turn superior to faces trained with negative expressions. Another possibility is that the meaning associated with each of the emotional categories is appraised. According to this hypothesis, identity recognition performance is determined by category of the expression rather than by its valence alone.

Studies have shown that facial expression has different effects on long-term and short-term face memory. In a short-term memory task such as face matching, performance is indistinguishable for faces displaying happy or disgust expressions (Levy and Bentin, 2008). Matching performance for happy faces is also comparable to that for faces with a sad or neutral expression (Chen et al., 2011). Similarly, immediate face recognition was not influenced by emotional expressions (i.e., smile, neutral, and anger) for 3 and 5 year old children (Freitag and Schwarzer, 2011). However, the effects can depend on the type of task. For example, more angry faces could be held in a working memory task (Jackson et al., 2009). In the present study, we have focused on the effect of expression on long-term memory. As noted earlier, the happy face advantage has been mainly demonstrated in long-term memory tasks, where participants are typically required to remember a series of faces in a training session, and later their recognition memory of the trained faces is measured in a test session. However, no study has compared all these basic facial expressions in a long-term memory task. Although Chen et al. (2011) compared effects of all basic facial expressions on identity recognition, they only tested short-term memory in a matching task. In the present study, because we employed the same set of stimuli in Chen et al. (2011), it was easier to make a comparison between the effects of the same expressions on long-term and short-term memory of face identities.

We used a standard old/new recognition task in this study. The effect of facial expression on identity recognition was assessed in two conditions, where the test faces either showed a same or a different expression from the trained faces. In the same-expression condition, the images of the target faces used at training and test were identical. In the different expression condition, the target faces changed from an emotional expression at training to a neutral expression at test. A change of facial expression results in a deformation of face shape and certain facial features. This can create a large image difference between the trained and test stimuli, which is known to impair recognition of unfamiliar faces (Bruce, 1982; Chen and Liu, 2009). After studying an image of an unfamiliar face, the observer’s ability to recognize the face again tends to depend on whether the same image of the face is presented. Recognition only becomes less image dependent when a face is well learned. By comparing the performance for the same-image and different-image conditions, we assessed whether some expressions at training could create a better transfer of training to a new image. The aim of this was to find out whether effects of expression on identity recognition facilitate image-invariant recognition. Our objectives were three fold. First, we aimed to verify whether the happy expression has an advantage in a face memory task over all other basic emotional expressions. Second, we investigated whether some other expressions enjoy a similar advantage although they may be less advantageous compared to the happy expression. And finally, we assessed whether some emotional expressions also transfer more effectively to a new expression (i.e., whether the studied faces with these expressions were relatively more recognizable in a different expression in the test session). Based on some of the prior studies reviewed earlier, we expected a superior identity recognition performance when faces were studied in a happy expression than in other expressions. Although the effects of some expressions such as disgust were not tested previously, we hypothesized they might also result in a poorer recognition performance relative to the happy expression because they all carry relatively greater negative valence.

## MATERIALS AND METHODS

### DESIGN

We employed a mixed design, where training expression (happiness, sadness, fear, surprise, anger, and disgust) was a between-participant variable, and test expression (same vs. different) a within-participant variable. Faces tested in the same expression were shown in identical images as the trained faces, whereas faces tested in a different expression were shown in a neutral expression. The dependent variable was *d′* scores that combined hits and false alarms.

### PARTICIPANTS

The study was approved by the Ethics Committee of the Department of Psychology, University of Hull. Written consent was obtained from each participant prior to the experiment. A total of 186 Caucasian students (127 female, 59 male) from the University of Hull took part in the study for course credit.

Each participant was randomly assigned to one of the six emotional expression groups (happiness, sadness, fear, surprise, anger, and disgust). Thus participants in the first group learned a set of faces with a happy expression, whereas participants in the second group learned the same faces with a sad expression, etc. To ensure that the effect of expression on identity recognition was due to expression alone, participants in different groups learned the same face identities but with different emotional expressions. Each group had 31–34 participants, who learnt faces in one of the assigned emotional expressions. The mean age of the sample was 23.1 years, SD = 7.3. The percentage of female participants in each group ranged from 60 to 77%. The median age in all groups was 21 years. All participants had normal or correct-to-normal vision.

### MATERIALS

The face database was obtained from Binghamton University, USA. It contained 100 3D faces and texture maps without facial hair or spectacles. Each 3D face file contained information about the spatial coordinates of points in a mesh that defined the surface shape of the face. Each such file was associated with a texture map, which is a photographic image that mapped the color at each point of the photograph onto the correspondent point of the surface in the 3D face file. The faces resembled real photographs when the texture map was applied to the surface model. The faces in the database were collected from the students, most in their early 20s, at Binghamton University. Some example faces are provided at the face database website: http://www.cs.binghamton.edu/∼lijun/Research/3DFE/3DFE_Analysis.html. More details about this database can be found in Yin et al. (2006). All models were shown in seven expressions (happiness, sadness, disgust, surprise, anger, fear, and neutral). Each emotional expression had four levels of intensity. Only the strongest intensity level was used in this study. We used all the 51 female Caucasian faces. We randomly chose 24 faces from this pool of faces for every six participants. Twelve of these were randomly assigned as targets, whereas the remaining faces were assigned as distractors. Each face model was rendered against a black background in the full frontal view (0°). The rendered faces were saved as gray-level bitmap images. The resolution of the images was 512 by 512 pixels.

### PROCEDURE

The recognition task consisted of a training session and a test session. Participants were informed of the two sessions and the tasks they would perform in each session. To enhance the effect of training and to prevent floor performance in the recognition task, we employed a face–name matching task in the training session following a similar procedure used in previous studies (Longmore et al., 2008; Liu et al., 2009). The face–name matching procedure required participants to pair names with faces. The repetition of the face images in this procedure allowed each trained face to be viewed several times before the recognition test took place. The required level of training was determined by a pilot study, which showed that each face should be shown at least three times during the training session to reach an overall accuracy of 73%, a recognition performance level between floor and ceiling.

In the initial block of the training session, 12 target faces were shown, one at a time for 5 s, after a 200 ms central fixation. A randomly assigned name was shown simultaneously with the face at the bottom of the screen. Participants were instructed to remember the face–name association. In the subsequent trials, each target face was again presented individually in the center of the screen for the same duration. This time the target face was accompanied by a row of four names at the bottom of the screen. One of the names was previously associated with the target, and the others were randomly chosen from the 12 names. The order of the four names was randomized. Participants were asked to choose the target face’s name by pressing one of the four corresponding keys. Feedback was given after each response. The correct answer was shown if a wrong name was chosen. The face–name matching task was repeated twice in two blocks of trials for the 12 targets.

The test session followed immediately after the training session. Instructions for this session were displayed on the screen. Participants were informed that the faces at the training session could display a different expression at the test session. In the test session, the 12 target faces were mixed with 12 distractor faces. Six of the targets were the same face images as they were at training and the other six targets switched to a neutral expression. The assignment of this was random. The 12 distractor faces mirrored the same assignment of expressions as the target faces in this session. The 24 test faces were shown one at a time, in the center of the screen, until the participant responded with a key press. To minimize the possibility of a recency effect, the presentation order of target faces during the testing session was the same as in the final set of the training session, with distractor faces randomly inserted into the sequence between targets. The alternation between targets and distractors followed the constraint that no more than three targets could be shown in a row. The names were not presented this time. Participants were instructed to judge whether the test face was seen at the training session. Participants responded by pressing one of the two keys labeled “Yes” or “No”. The test face remained on screen until a response was made.

## RESULTS

Data from 15 participants were excluded due to their overall chance level performance in the test session (50%). Four of these participants were from the anger group, four from the fear group, two from the happiness group, two from the sadness group, two from the disgust group, and one from the surprise group. Data from the remaining 171 participants, whose overall performance was 71% (SD = 9.6) for the session, were used for further analyses.

### RESULTS OF FACE–NAME MATCHING TASK

The percentage accuracy data of this task in the training session was analyzed using the two face–name matching blocks as a within-participant variable, and the training expressions as a between-participant variable. A repeated-measures analysis of variance (ANOVA) on Arcsine square root transformed percentage correct response showed a significant main effect of training *F*(1,165) = 3.66, *p* < 0.05, $\eta_{p}^{2}$ = 0.02, where performance in the second block (*M* = 50.9%, SD = 19.5) was better than the first block (*M* = 48.7%, SD = 18.5). There was no difference between results of training expressions, *F*(5,165) = 0.41, *p* = 0.84, $\eta_{p}^{2}$ = 0.01, where the mean scores were 51.8% for happiness (SD = 18.7), 51.7% for fear (SD = 17.8), 50.6% for anger (SD = 16.7), 48.4% for sadness (SD = 14.1), 48.3% for surprise (SD = 13.7) and 46.5% for disgust (SD = 15.6). No significant interaction was found between the two factors, *F*(5,165) = 0.41, *p* = 0.84, $\eta_{p}^{2}$ = 0.01.

### RESULTS OF RECOGNITION TASK

**FIGURE 1:**
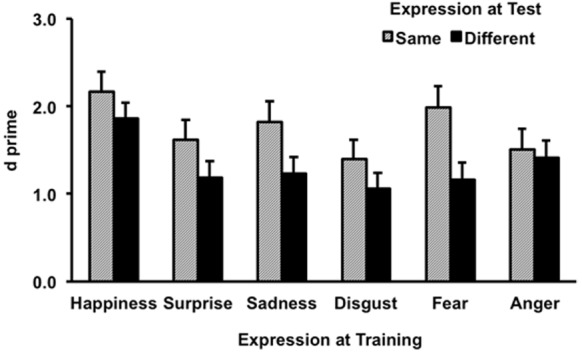
**Sensitivity results as a function of trained and tested expression.** Error bars represent one standard error above the means.

Results for hits, correct rejections, and the combinations of these in the test session are shown in Table 1. Statistical analyses were applied to *d′* and criterion (c) data derived from the results of hit (H) and false alarm (FA) rates, where *d′* = *z*(*H*)–*z*(FA), and *c* = –[*z*(*H*) + *z*(FA)]/2. The *d′* results are shown in Figure 1. An ANOVA on the *d′* data revealed a significant main effect of expression change, *F*(1,165) = 3.34, *p* < 0.001, $\eta_{p}^{2}$ = 0.08, where performance for same expression was better than different expression. There was also a main effect of expression, *F*(5,165) = 3.13, *p* < 0.01, $\eta_{p}^{2}$ = 0.09. No significant interaction was found between these factors, *F*(5,165) = 0.73, *p* = 0.60, $\eta_{p}^{2}$ = 0.02.

**Table 1:** Mean percent correct responses as a function of trained expression and expression change.

	**Tested expression**					
	**Same**			**Different**		
**Learned expression**	**Hit**	**CR**	**Overall**	**Hit**	**CR**	**Overall**
Happiness	82.2 (15.1)	74.4 (24.7)	78.3 (15.1)	66.7 (24.4)	80.0 (22.1)	73.3 (16.0)
Surprise	73.6 (18.1)	70.7 (24.3)	72.1 (15.3)	57.5 (19.2)	78.7 (15.4)	65.0 (13.6)
Sadness	82.7 (17.3)	64.9 (23.8)	73.8 (14.5)	58.3 (15.4)	78.6 (15.6)	68.5 (9.4)
Disgust	83.9 (14.2)	52.8 (23.2)	68.3 (11.2)	56.1 (19.8)	73.9 (19.4)	68.1 (9.2)
Fear	82.1 (15.3)	73.5 (17.5)	77.8 (11.8)	50.0 (25.3)	79.0 (20.5)	64.5 (14.2)
Anger	75.3 (19.2)	66.7 (20.1)	71.0 (13.1)	59.3 (24.6)	79.0 (18.8)	69.1 (12.4)

*Note: values in parentheses represent standard deviations*.

*CR, correct rejection; overall, combined accuracy of hit and correct rejection*.

The Tukey honest significant difference (HSD) posteriori tests revealed a significantly better recognition performance for happiness than for disgust (*p* = 0.003). Other pairwise comparisons did not yield significant difference (*p*s > 0.05).

**FIGURE 2:**
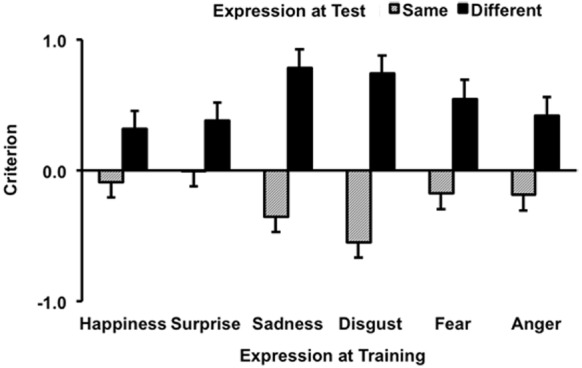
**Criterion results as a function of trained and tested expression.** Error bars represent one standard error above the means.

Results of criterion data are shown in Figure 2. A liberal response is indexed by a negative value, whereas a conservative response by positive. ANOVA showed a significant main effects of expression change, *F*(1,165) = 103.24, *p* < 0.001, $\eta_{p}^{2}$ = 0.39, where response criterion for different expression was more conservative than for same expression. The main effect of expression was not significant, *F*(5,165) = 0.29, *p* = 0.92, $\eta_{p}^{2}$ = 0.01. However, this was qualified by a significant interaction between the two factors, *F*(5,165) = 4.46, *p* < 0.001, $\eta_{p}^{2}$ = 0.12.

Simple main effect analyses revealed no significant main effect of expression when the test face had a different expression, *F*(5,165) = 1.86, *p* = 0.11. However, the main effect of expression was significant when the test face showed the same expression as the trained face, *F*(5,165) = 2.96, *p* < 0.01. Tukey HSD comparisons showed response criterion for disgust was more liberal than for surprise (*p* = 0.013). All other pair-wise comparisons were not significant (*p*s > 0.06). We also analyzed the simple main effects of expression change for each individual expression. This revealed a significant difference for every expression, all *F*s > 6.70, *p*s < 0.01. However, it is evident from Figure 2 that expression change modulated response criterion more strongly for some expressions than others, which was likely a cause of the significant interaction. We computed the difference of response criterion for each expression by subtracting the mean for different expression from the mean for same expression. The difference scores were then submitted to an ANOVA, which yielded significant effect, *F*(5,165) = 4.46, *p* < 0.001. Pair-wise comparison showed a significant difference between happiness and disgust (*p* = 0.008), and surprise and disgust (*p* = 0.006), where criterions in the happy and surprise conditions were less affected by the modulation of expression change. All other pair-wise comparisons were not significant (*p*s ≥ 0.06).

### DISCUSSION

This study aimed to verify whether the happy expression has an advantage in a face memory task over other basic emotional expressions and whether any other expressions enjoy a similar advantage. It also aimed to assess whether recognition memory of faces learnt through some emotional expressions was more robust against an expression change. Our results demonstrate a significant influence of training expression on identity recognition. When the effects of the six expression training conditions were compared, we found a better identity recognition performance for faces trained in a happy expression than those trained in a disgust expression. However, the result in the happy face condition was not more superior to other expression conditions. No difference was found among recognition accuracies for other expression conditions. The results thus provide only partial support for the hypothesis that the happy expression produces better identity recognition memory relative to other facial expressions. Our data also show that transfer from an emotional expression to a neutral expression impaired recognition performance relative to the same-expression condition, where images used for training and test were identical. This replicates the typical finding that recognition of unfamiliar faces is image dependent. Since the effect of training expression on recognition accuracy did not interact with the effect of expression change, it is likely that the training expression affected the two testing conditions equally, regardless of whether the trained faces were tested in an identical image or a new image.

Facial expression also had a significant influence on response criterion when the test face showed the same expression as the trained face. However, only the difference between the results of disgust and surprise reached a level of significance, where response for disgust was found to be more liberal than for surprise. It is not surprising that when faces were trained and tested in a different expression, participants tended to respond more conservatively than when they saw the same trained expression/images in the test session. However, it is unclear why response criterion for surprise was the least affected, whereas disgust was most affected by this trend in the same image condition. The criterion results (Figure 2) show that the six training expressions were differentially modulated by the change of expression in the test session. Disgust and sadness were especially sensitive to the modulation, although only the disgust condition had a significantly larger difference between criteria for same and different expressions than some of the other training expressions, such as happiness and surprise.

Although the overall finding in our study is consistent with the happy-face advantage reported in the literature, some of the previous findings were not replicated. Among these are the benefits of happy expression for identity recognition over surprise, anger, and fear (Endo et al., 1992; Baudouin et al., 2000; Kaufmann and Schweinberger, 2004; Gallegos and Tranel, 2005; Shimamura et al., 2006; D’Argembeau and Van der Linden, 2007, 2011). Our results for the happy expression were not significantly different from these expressions. Nonetheless, given the trends suggested in the means for these conditions (Figure 1), our results are not in contradiction with the previous findings. The lack of significant differences could be attributed to the limitation of the between-participant design, which introduced variance due to individual differences as indicated by the relatively large standard deviations (Table 1). Most other studies have employed a within-participant design, which might make it easier to detect an effect. There are also notable differences in other aspects of methods that could have contributed to the disparate findings. For example, the task during the training session in D’Argembeau and Van der Linden (2007) required judging intelligence/nose size/expression intensity of the trained faces, whereas the task during this session in our study required remembering the names of the trained faces. Different tasks at the time of training could modulate the effect of facial expression. Needless to say, the use of faces from different databases could be a factor as well. Future research will need to discriminate the factors that determine the extent to which facial expressions can have an effect on identity recognition.

Faces with a disgust expression produced worst mean accuracy of recognition memory. The effect of this expression on long-term memory was not studied previously. However, a similar effect was found in a short-term memory task: along with the angry expression, disgust also produced poorest matching performance in both accuracy and response time (Chen et al., 2011). A disgust or angry expression made a face identity more difficult to match, regardless of whether the task required matching faces in the same expression or different expressions. Although memory for angry faces was not significantly worse than for happy faces in our study, the level of performance for angry faces was similar to that for disgusted faces. However, there is also an important difference between the results of these tasks. In the present recognition task, the happy expression condition was the only one that produced superior recognition performance to the disgust expression. In the matching task reported in the previous study, however, some expression conditions (such as sadness and fear) other than the happy-expression condition were also matched more accurately than those showing a disgust or angry expression. Chen et al. (2011) pointed out that the difference between the results of these tasks could mean that happy faces linger longer in memory, although perceptually they are not necessarily easier to match than faces with some other expressions.

It remains difficult to explain why faces displaying a disgust or angry expression are harder to identify or to match. Based on some speculations, an angry expression directs attention to an emotional situation, which reduces resource for identity processing (D’Argembeau and Van der Linden, 2007). The same may be true of a disgust expression. Perhaps other expressions demand less resources of attention. Indeed, research has shown that the happy expression is processed more efficiently than other expressions. For example, when participants were required to judge the expression of a briefly presented face, they consistently identified happy faces faster and more accurately (Calvo and Lundqvist, 2008). A happy face is also detected more quickly from an array of faces (e.g., Juth et al., 2005; Calvo and Nummenmaa, 2008). These studies show that processing for other expressions is relatively more demanding. Perhaps when expressions are processed less efficiently, they tend to compete with identity processing for attentional resources. This may explain why the disgust expression impaired identity recognition in the present study. However, this explanation will require careful scrutiny because if the effect in our study could be explained by the efficiency of expression processing, then it is not clear why the fearful expression did not also impair identity recognition. Some studies showed that fear rather than disgust was the least efficiently processed expression (Calvo and Lundqvist, 2008; Calvo and Nummenmaa, 2008). However, a later study (Calvo and Nummenmaa, 2011) did find that disgust and surprise were discriminated least efficiently. This would be consistent with the explanation of our finding based on the role of processing efficiency.

The distinct effects of happy and disgust expressions may depend on the nature of face perception tasks. Although these expressions produced a difference in our identity recognition task, they had no measureable effect on the face–name matching task in the training session. This may indicate that retrieving the name of a face is less affected by the distribution of attentional resources. However, there is little doubt that the recognition task in our study demonstrates an effect of encoding emotional faces on identity recognition. Recognition memory of face identities was clearly affected by facial expressions of the trained faces, although the facial expressions were task irrelevant. This shows that the identity and expression of a face are not processed by two completely independent routes as was originally proposed in Bruce and Young’s (1986) classical model of face perception. Our study is in agreement with the conclusion that the separate route theory is less well supported (e.g., Calder and Young, 2005; D’Argembeau and Van der Linden, 2007, 2011).

The pattern of the results in the present study is not easily explained by predictions based on an appraisal of emotional valence alone. The valence of surprise can be more neutral, or less negative relative to some of the expressions such as sadness, fear, and anger, yet there is no indication that faces with a surprise expression could be remembered more accurately than faces with negative expressions. However, it would be simplistic to dismiss the valence prediction based on the lack of significant difference between these conditions.

A similar mixed picture for the valence account was reported in prior research on effects of expression in face matching (Chen and Liu, 2009). Since happiness produced better matching performance than expressions of disgust and anger, results in Chen et al. (2011) fit with the valence account based on the contrast of positive vs. negative emotions. However, the researchers found it difficult to explain why the happy expression was not also more effective in producing better matching performance than other negative expressions such as sadness or fear. Given the common pattern of results from the present and previous studies, it is possible that recognition performance is affected by appraisals of specific content of an expression rather than by a simple dichotomous valence metric alone.

Some studies have shown that the benefit of emotional stimuli relative to a neutral one on memory is driven by both the valence of stimuli and the arousal intensity of induced emotion (LaBar and Cabeza, 2006). For example, the arousal value of a fearful expression may be higher relative to happy and neutral stimuli (Morel et al., 2009). It is possible that memory processes are related specifically to emotionally arousing faces. Since the present study only used the highest intensity for all expressions, we were not able to evaluate this possibility. The potential interaction between the effects of expression and emotional intensity can be a meaningful question for future research.

In summary, this study reveals a clear influence of facial expression on identity recognition. The main difference between our study and the previous literature was that we compared effects of all six basic emotional expressions on recognition memory under the same controlled condition that separated identity-related shape information from expression-related shape information. In addition to the existing knowledge, we found that the disgusted expression was especially detrimental to identity recognition. Our results also reveal similarities and differences between the effects of expression on long-term and short-term memory tasks. We found that the happy expression has negligible effect for a face–name matching task, but its advantage starts to emerge for identity recognition after the expression and the identity information are co-registered in long-term memory.

### Conflict of Interest Statement

The authors declare that the research was conducted in the absence of any commercial or financial relationships that could be construed as a potential conflict of interest.
